# Translation, adaptation, and initial evaluation of a guided self-help intervention to reduce psychological distress among nurses during COVID-19 in China

**DOI:** 10.3389/fpsyt.2023.1168117

**Published:** 2023-08-17

**Authors:** Tian Tian, Jingwen Sun, Yue Jiang, Qian Guo, Zeyu Huang, Duolao Wang, Atif Rahman, Xiaomei Li, Lei Yang

**Affiliations:** ^1^School of Nursing, Health Science Center, Xi'an Jiaotong University, Xi'an, China; ^2^Department of Clinical Sciences, Liverpool School of Tropical Medicine, England, United Kingdom; ^3^Department of Psychological Sciences, University of Liverpool, England, United Kingdom

**Keywords:** psychological distress, nurses, self-help plus (SH+), COVID-19, pilot project, cultural adaptation

## Abstract

**Background:**

This study aimed to reduce the unprecedented and intense psychological distress that nurses were forced to experience during the COVID-19 pandemic. A Chinese version of the World Health Organization's Self-Help Plus (SH+) intervention guide was adapted and tested among nurses. The objective of this study was to translate and adapt the SH+ guideline into the Chinese version and to test its feasibility in reducing psychological distress among nurses during COVID-19.

**Methods:**

A staged approach comprising translation, adaptations, initial evaluation by pilot implementation, and a qualitative process evaluation was conducted in two hospitals in Xi'an, China. The translation of the Chinese version was authorized by the World Health Organization. We adapted SH+ for use among clinical nurses working during the pandemic in China through a qualitative process evaluation, which was guided by the descriptive phenomenological study design. The outcomes of the pilot included psychological distress, psychological flexibility, depressive and anxiety symptoms, post-traumatic stress disorder (PTSD) symptoms, and subjective psychological wellbeing, which were assessed using the Kessler 6 symptom checklist, the Comprehensive Assessment of ACT Process (CompACT), the Patient Health Questionnaire-9 (PHQ-9), the Generalized Anxiety Disorder scale (GAD-7), the PTSD Checklist-Civilian Version (PCL-C), and the Index of Wellbeing (IWB), respectively.

**Results:**

The SH+ materials, including audio-recorded sessions and an accompanying illustrated manual, were translated into Chinese and adapted in line with feedback from the nurses. An uncontrolled pilot study (*n* = 28) for 5 weeks showed a statistically significant reduction of psychological distress (mean difference in Kessler 6 score, −2.74; 95% CI [−3.71, −1.78]; *p* < 0.001). We also found improvements in psychological flexibility (mean difference in CompACT score, 6.89; 95% CI [−12.35, −4.47]; *p* < 0.001), subjective psychological wellbeing (mean difference in IWB score, 0.86; 95% CI [0.07, 1.65]; *p* < 0.05), and depressive symptoms (mean difference in PHQ-9 score, −1.52; 95% CI [−2.78, −0.26]; *p* < 0.05). The process evaluation showed that nurses found the SH+ program very useful but difficult to adhere to.

**Conclusion:**

We found that the translated Chinese version of SH+ was applicable and feasible in the Chinese cultural context. There was a potential effect of adapted SH + in reducing nurses' psychological distress during COVID-19 and suggested the value of exploring strategies to increase adherence to the program.

## 1. Introduction

The outbreak of COVID-19 has posed a serious public health threat worldwide in the past 3 years. Studies found that with a high rate of infection and death, COVID-19 led to moderate-to-severe psychological problems, including psychological distress, anxiety, depression, fear, psychosomatic preoccupations, and insomnia in the general public (1, 2). Facing this critical situation, the nurses were one of the groups most severely affected by the COVID-19 pandemic, leaving them with serious psychological effects, given their risk of exposure to the virus, concerns about infecting and caring for their loved ones, longer work hours, and shortages of personal protective equipment (1, 3). Studies reported that a considerable proportion of nurses were experiencing high levels of depression, anxiety, psychological distress, post-traumatic symptoms, burnout, and insomnia (4–6). A systematic review showed that the pooled prevalence rate of psychological distress among nurses during the COVID-19 outbreak was 46.1% (7). Despite the passage of time after the epidemic began, there were still psychological repercussions for nurses as a result of increased labor and exhaustion (3, 8), which highlighted the need for designing a targeted intervention to improve mental health and foster post-traumatic recovery among nurses.

Psychological distress is a response to specific stressors or demands and is characterized by a perceived inability to effectively cope with the stressors, a change in emotional state (such as stress, anxiety, and depression), and an expression of discomfort that causes either temporary or permanent harm to the person (9). The cost of psychological distress among nurses is high since it can result in fatigue, impoliteness, anxiety, an increase in blood pressure, a lack of self-confidence, and a decline in productivity (10). Consequently, it is imperative to develop strategies to promote the mental health and wellbeing of nurses throughout the COVID-19 pandemic and beyond.

However, multiple barriers are limiting the implementation and ability of current conventional interventions for the management of distress in nurses. Evidence-based interventions for nurses' psychological distress remain scarce in the literature (11). Moreover, it is hard to deliver long-term universal psychological care to nurses due to shift hours, an overload of work commitments, and a lack of time to attend sessions. Traditional face-to-face psychotherapy is particularly hard to implement immediately and regularly for nurses working in the context of quarantine policy due to the pandemic. Additionally, traditional psychotherapy interventions generally require a substantial clinical workforce, such as mental health specialists and specialist facilities, which are especially not available in developing countries (12). Furthermore, recent experiences in China demonstrate that not all nurses willingly partake in group or individual psychological interventions due to the stigma surrounding mental health issues (13).

Given these challenges, urgent attention must be paid to the exploration of feasible strategies to enhance nurses' access to evidence-based psychological interventions during the COVID-19 pandemic. In an effort to make available a series of scalable psychological interventions, the WHO and a number of institutions have proposed guidelines for the provision of psychological assistance to healthcare workers during this pandemic (14). The Self-Help Plus (SH+) program is a guided, multi-media, self-help intervention that is part of the WHO's flagship mental health gap action program (mhGAP). SH+ is a low-intensity psychological intervention for stress management and overcoming a variety of adversities (15) and is informed by various meta-analyses of its therapeutic ingredients. It has been translated into several languages (Spanish, Arabic, French, Greek, Japanese, etc.) and has been implemented in many countries (16). The intervention is founded on the principles of Acceptance and Commitment Therapy (ACT), a third-wave cognitive-behavioral therapy intended to increase psychological flexibility (17). Evidence has shown that ACT has promising effects on stress, anxiety, depressive symptoms, and quality of life (18). The central construct of ACT is psychological flexibility (PF), which is defined as an individual's ‘ability to contact the present moment more fully as a conscious human being and to change or persist in behavior when doing so serves valued ends' (17). Studies have shown that ACT is a suitable intervention in a self-help format, particularly when clinician guidance is given (19). Compared to conventional psychotherapy, the guided self-help ACT approaches were cheaper and easily accessible and offered a feasible alternative to resource-constrained psychotherapeutic interventions (19, 20). Given these findings, we expected that adaptations would be required to enhance acceptability, feasibility, and satisfaction with the intervention in this socio-cultural context.

In this article, we describe the translation, adaptation, and initial evaluation of SH+ with nurses during COVID-19 in China. The purpose of this research was to adapt SH+ for the Chinese population and to determine the acceptability, comprehensibility, and cultural Relevance of the guided self-help model for reducing psychological distress in nurses.

## 2. Materials and methods

### 2.1. Study design

An approach with three phases for translation, adaptation, and piloting was utilized, including (1) translation and adaptations; (2) pilot implementation; and (3) process evaluation.

#### 2.1.1. Phase 1: translation and adaptations

For this phase, the translation and adaptations of the WHO-SH+ manual, as well as the handbook and audio materials, were completed. Translation and adaptations were conducted in three steps: (a) translation by a team of bilingual researchers; (b) adaptation by an expert group; and (c) pre-testing and cognitive interviewing.

The translation and adaptations of SH+ intervention materials (guidelines, illustrations, and recordings) were guided by the Bernal framework of translation and adaptation of interventions, which includes eight dimensions: language, persons, metaphors, content, concepts, goals, methods, and context (21). Not only does the framework provide a useful documentation method, but it also permits translators and experts to concentrate on the key dimensions that need to be adapted (22). Cognitive interviewing was used to conduct a pre-test that guided the adaptation process. Cognitive interviewing is a common technique for validating the accuracy of health questionnaires or interventions created in one cultural context and then implemented in another language and culture (23).

##### 2.1.1.1. Translation

The original English version of the SH+ manual was provided by the World Health Organization (16), and permission was obtained to translate and adapt it into the Chinese context. The translation process was conducted by three investigators (XL, LY, and TT, native Chinese), led by a psychological nursing professional (XL). A forward translation of the complete SH+ materials was produced by two translators (LY and TT). Then, the translation was reviewed and edited by a senior translator (XL).

##### 2.1.1.2. Adaptations

The adaptations were conducted through a face-to-face group meeting and an online meeting with a group of mental health experts/mental health professionals (four per group) by facilitators. Experts from the professional group were from different areas, including two psychological nursing professionals (XL and JL) and two experts from the Institute of Psychology, Chinese Academy of Science (ZL and RW). The facilitators (ZH and JS) were members of the research team who were familiar with the SH+ manual and the procedures for its adaptation and piloting. Facilitators' responsibilities included (a) briefing the participants and organizing groups to work more effectively; (b) supporting the intervention process and monitoring the intervention sessions received by the participants during piloting; and (c) reporting the process.

Through discussions of any problematic items with the expert group, consistency was achieved at both the technical level (i.e., wording, grammar, tense, punctuation, and the acceptable level of abstraction) and the conceptual level (obtaining an identical meaning of concepts that may have different cultural expressions, such as idioms and metaphors). In addition to revising the cultural adaptation of the language and illustrations, the expert group suggested the context and conditions for program delivery. In addition, parts of the illustrations in the SH+ manual were modified or re-drawn by two Chinese artists to suit the Chinese context (e.g., changing characters, styles of clothing, dressing up, and environment).

##### 2.1.1.3. Cognitive interviews

The cognitive interview approach was applied to gain the perspectives of the users, i.e., nurses, to guide further adaptation. A total of 14 nurses of both sexes and various ages from the First Affiliated Hospital of Xi'an Jiaotong University who have experience in caring for infected patients or have performed other related work in the quarantine area during the COVID-19 pandemic participated in interviews. They were divided into groups of 3–4 participants. We asked them to read the handbook, listen to the audio, and watch the exercise video individually. Following this, the facilitators obtained their feedback and noted any concerns they might have. A structured questionnaire regarding comprehensibility, acceptability, relevance, and any proposed changes was administered. Finally, any potential changes were summarized in a structured form, discussed by the research team, and incorporated into the adapted manual and audiovisual material.

Detailed instructions for the cognitive interview are provided in Appendix 1.

#### 2.1.2. Phase 2: pilot implementation

The pilot study was designed as a one-arm intervention study without a control group.

##### 2.1.2.1. Participants

The pilot study was conducted at the Second Affiliated Hospital of Xi'an Jiaotong University. Nurses who were on duty from 20 October 2020 to 27 November 2020 were eligible for this study. Twenty-eight staff nurses in the hospital were recruited for the pilot study. The inclusion criteria of the pilot study were as follows: (1) nurses who scored 5 or above (moderate psychological distress) on the Kessler Psychological Distress scale (K6); (2) consent to participate in this study and signing a written informed consent; and (3) have taken care of patients during the pandemic. The exclusion criterion was severe mental disorders or imminent risk of suicide which was assessed by the K6 scale. Those scoring above 13 on the K6 scale were reassessed with the assistance of a psychiatrist to determine exclusion from the study (24).

##### 2.1.2.2. Measures

The 6-item Kessler psychological distress scale (K6) (25), a simple measure to identify levels of distress, was used as the primary outcome measurement, which was measured at baseline and within 5 weeks post-intervention. The secondary outcomes included psychological flexibility, depressive and anxiety symptoms, post-traumatic stress disorder (PTSD) symptoms, and subjective psychological wellbeing, which were assessed by the Comprehensive Assessment of ACT Process (CompACT) (26), the Patient Health Questionnaire (PHQ-9) (27), the 7-item Generalized Anxiety Disorder Scale (GAD-7) (28), the PTSD Checklist-Civilian Version (PCL-C) (29), and the Index of Wellbeing (IWB), respectively. All the measures used in the study have been translated and adapted into Chinese versions.

##### 2.1.2.3. Intervention

The intervention used the adapted Chinese version of the SH+ package, including a package of pre-recorded exercises and an illustrated self-help manual with five parts: grounding, unhooking, acting on your value, being kind, and making room. The intervention was conducted through an online WeChat mini program for 5 weeks. Before the first SH+ intervention session, all participants were introduced to the contents and mode of intervention by the facilitators in a face-to-face session and reminded afterward in an online session. The five-session pre-recorded audio and video material was pushed via the WeChat mini program and also delivered to a WeChat group of 28 nurses in 5 weeks. The audio material imparted key information about stress management and guided the participants through individual exercises. To augment the course materials, an illustrated self-help course was presented to review all essential contents and concepts, and additional videos provided complementary material to aid understanding. A session reporting form was completed after each intervention session by the facilitators.

#### 2.1.3. Phase 3: a process evaluation

A descriptive phenomenology approach was used for the process evaluation, in which a semi-structured interview was conducted among 28 participating nurses and facilitators to explore their experience and suggestions for the implementation of the program. Descriptive phenomenology, represented by Edmund Husserl, is an approach that emphasizes “To the things themselves” to depict the real world, to make people listen to phenomena more fully and truthfully, to stimulate people's feelings and observations of everyday experiences, and to increase the depth, width, and breadth of these experiences (30, 31). Thus, guided by the methodological approach, the researchers remain open and fully immersed in the research phenomenon throughout the entire research process in order to obtain an accurate description of the participant's experience in the program.

Two researchers (TT and ZY, H) conducted a semi-structured one-on-one telephone interview with 28 nurses, focusing on program perceptions, frequency of use, helpfulness, appropriateness, most valuable components, barriers to the adoption of the intervention, and recommendations for subsequent application. In addition, two facilitators were interviewed on the biggest challenges in training and supervising program implementation.

As the interviewees were divided into two groups: nurses and facilitators, the analysis of the qualitative data was carried out by two researchers who were each initially responsible for one group of data analysis, and they exchanged with each other to check the final result with key informants to ensure that the final presentation of the data accurately reflects the experience. In addition to this, the process of the data analysis and all significant statements were examined and validated by an expert researcher (XL) to ensure the correctness of these processes, the consistency of the meanings, and the accuracy of the overall thematic map.

### 2.2. Data analysis

Descriptive statistics were used to describe the socio-demographic characteristics of the sample at baseline. The bivariate Pearson's correlations were tested between the average sum of scores on scales. The pre-and post-assessment measures were compared using a paired samples *t*-test to examine sensitivity to change and to analyze the general direction of changes before and after the intervention, after validating the assumptions of the paired samples *t*-test. Statistical analyses were carried out using SPSS 19.0.

A descriptive phenomenological approach using Colaizzi's seven-step data analysis within the phenomenological empirical framework was used for qualitative data. Colaizzi's unique seven-step process provides a rigorous analysis, with each step approaching data and widely used in disciplines such as health science (32, 33). The two researchers collected the data verbatim by transcribing the interviews in Chinese within 24 h after the interviews. The following seven-step approach was adopted: (1) Familiarity: they became familiar with the data by reading through all of the participant accounts several times. (2) Identifying significant statements: they identified all statements in the accounts that were directly related to participants' perspectives, program obstacles, and expectations. (3) Formulating meanings: they encoded significant statements and labeled them with the participants' keywords and phrases. (4) Clustering themes: they clustered the identified meanings into themes that are common to all accounts. (5) Developing an exhaustive description: they provided a detailed description of the resulting themes, incorporating all the themes generated in step 4. (6) Developing the basic structure: they identified and extracted similar ideas by repeatedly comparing similar themes and descriptions, and they built short and dense meaningful themes. (7) Seeking verification of the basic structure: they returned the basic structure declaration to all participants and asked if it reflected their experience.

### 2.3. Ethical conduct of research

The study was approved by the Ethics Committee of the Health Science Center at Xi'an Jiaotong University (No. 2020–1332). Written informed consent has been obtained from the participants.

## 3. Results

### 3.1. Phase 1: translation, adaptations, and cognitive interviews

#### 3.1.1. Translation and adaptations

The intervention's fundamental structure, concepts, and techniques were culturally compatible and did not require significant modifications during the translation process. Participants from the nurses' group mentioned that most parts of the illustrated manual were comprehensible and acceptable, and helped reduce distress from working in the COVID-19 context. Nonetheless, subtle but essential adjustments were required in several domains. Key areas and examples of adaptations are given in Table 1.

**Table 1:** Bernal framework of adaptations and examples of key adaptations.

**Dimension**	**Operationalization**	Examples of key adaptations^*^
Language	Emotional expression, verbal style	•All the materials were translated into simplified Mandarin. •The language was kept specifically colloquial rather than formal. Translations were conceptual rather than literal and word-to-word, to make the participants understand the underlying ideas and the concepts of the SH+.
Persons	Facilitators and the client – counselor relationship	•Facilitators were identified as acceptable delivery agents as they have ethnic, racial, and professional similarities with the client.
Metaphors	Symbols and concepts, sayings / proverbs	•The addition of the common Chinese proverb “*A journey of a thousand miles begins with a single step”* from <Tao Te Ching> elaborated on the importance of persistence. The metaphor was that the key to relieving distress is gradually accumulated from small to large, from little to too much via practicing. •The addition of Chinese idioms like “*A single spark can start a prairie fire”*–implied that the more we practice noticing our thoughts and feelings and refocusing on what we are doing, the better we will get. •Images embodying the nurse's avatar were used.
Content	Familiarity with local values, customs, traditions, and nursing context	•Replace expressions and scenarios that do not fit the Chinese condition,e.g., changing the “violence in the community” and images in exotic costume. •The addition of nursing contexts. •Examples of the stressors that were culturally and professionally suitable, e.g., conflicts between working and the family, occupational stresses during the COVID-19 were added.
Concepts	Constructs of theoretical model – how nurses' problems was perceived and communicated, including availability of locally used terms for theoretical concepts	•Personal concept (e.g., the values of “*prudence and professionalism”* and “*superb skill or technique”* according to traditional Chinese concept of pursuing excellence, and striving for perfection in one's life, and emphasizes rising in great vigor in one's work.) were added. •Social concept (e.g., “*The benevolent person always cares for others.”*) were added.
Goals	Reflecting knowledge of values, culture, customs and traditions	•To encourage active participation of the nurses, the additional interview of the introduction to the content was added.
Methods	Methods and procedures to deliver the intervention	•Every part of the five sections of the self-help guide was given to nurses via WeChat each week, divided into five weeks to complete. Focused group interviews were organized before and one after the intervention. •Several ways of getting self-help activities, including audio, video and text online and off-line materials were given to nurses to choose.
Context	The culturally sensitive element of the context including nurses' distress during the pandemic, Chinese developmental stages, and availability of psychological supports for nurses	•All the SH+ materials were modified for integration into the Chinese context. •Some face-to-face SH+ sessions were abbreviated and integrated to match the context of general isolation and control in the hospital during the pandemic of the COVID-19.

**Figure 1:**
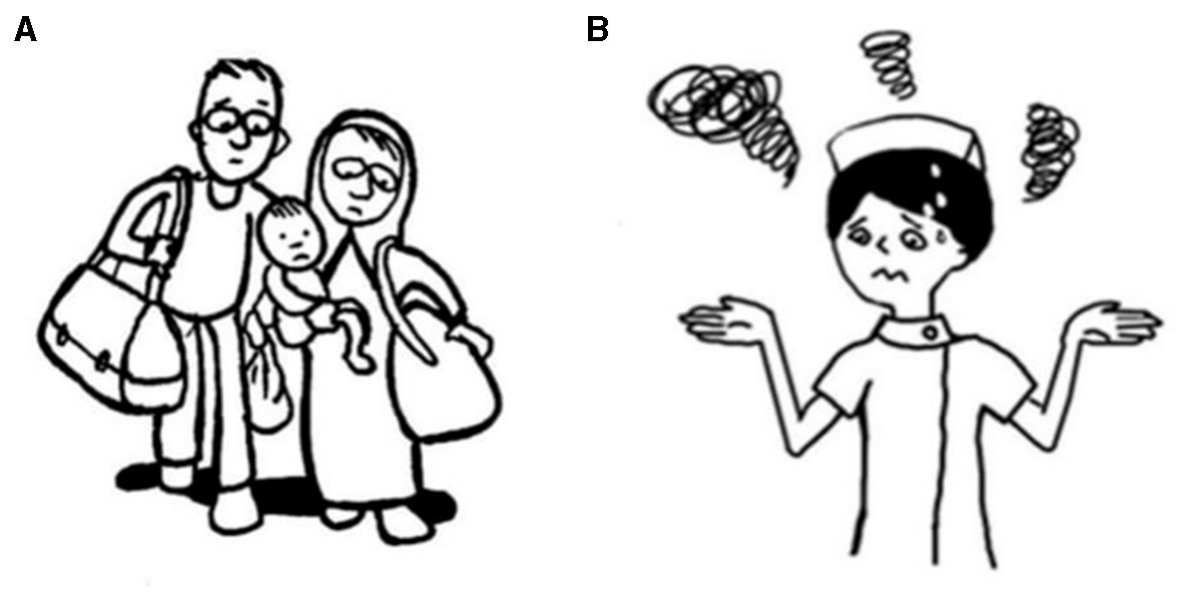
An example of the adaptation of characters in the content dimension. **(A)** is a family displaced from home in the original context in the original context and **(B)** is the adapted image of a nurse worrying about the change of daily life rhythm during the pandemic of COVID-19.

In accordance with Bernal's framework, the translator sought to translate the conceptual equivalent of a phrase, as opposed to a literal translation, while keeping the target audience in mind. (i) *Language*: By avoiding long and convoluted sentences and specialized terminologies and jargon, the language was kept simple, plain, and concise. (ii) *Persons*: Facilitators providing the intervention implementation were perceived to be perfectly acceptable as delivery agents during COVID-19, given their role in the promotion of mental health for nurses. (iii) *Metaphors*: Several culturally relevant metaphors were employed to increase nurses' understanding and motivation and to reinforce the cultural context of the content. (iv) The *content* of the intervention material was adjusted to suit China's national conditions and customs. Similarly, the content was modified to use characters and scenarios based on the nursing context, including nursing situations during COVID-19. For example, the distress that nurses faced was incorporated (Figure 1). (v) The *concepts* of society and culture were examined carefully and adapted. The Chinese nation inherits self-improvement, so the value concept of honesty and professional dedication was incorporated. At the same time, several mainstream social values in China, such as “kindness”, “caring”, and “harmony”, were also added to the content. (vi) *Goals*: With many cultural and traditional differences in mind, a greater emphasis was placed on client-derived objectives as opposed to prescriptive goals. (vii) *Methods*: Some pictures were re-drawn to match the Chinese settings and culture. The delivery of the intervention was made compatible with the way nurses got used to it. (viii) *Context*: Adaptations to the context were necessary to ensure that the intervention could be implemented with the existing healthcare systems.

#### 3.1.2. Cognitive interviews

In the cognitive interviews with clinical nurses, we found that further improvements were suggested by the end users. In terms of the self-help contents, it was generally understandable, although some nurses indicated that a few chapters and words were abstract and difficult to understand (e.g., “Imagine Gas flows in and around this object”). Furthermore, the subtitles also needed to be simplified and popularized to attract the attention of the participants. In addition, the brief introduction before each chapter was also highly recommended by nurses to meet their needs for a brief understanding of the learning purpose and then in-depth study and reading.

### 3.2. Phase 2: pilot implementation

#### 3.2.1. Demographic characteristics at baseline

A total of 28 nurses with K6 scores above 5 were recruited for the pilot study. All of them were women who were aged between 24 and 54 years (34.48 ± 5.81). Close to nine-tenths (89%) were married, and 12 nurses had a bachelor's degree or higher education level. Further characteristics of the participants are presented in Table 2.

**Table 2:** Demographic characteristics of the pilot sample at pre-assessment (*n* = 28).

**Characteristics**	***n* (%)**
**Age** (year)	34.48 (5.81)
**Years of work** (year)	11.74 (5.93)
**Marital status**	
Never married (single)	3 (11)
Married	25 (89)
**Family income (per capita CNY)**	
5000	5 (18)
5001–10000	14 (50)
10001–15000	6 (21)
15001	3 (11)
**Education level**	
Technical secondary school	2 (8)
Associate degree	14 (50)
Bachelor's degree or above	12 (42)
**Professional title**	
Nurse	1 (4)
Nurse practitioner	11 (39)
Nurse practitioner in charge/Supervisor nurse	14 (50)
Chief nurse practitioner or above	2 (7)
**Position**	
Nurse	17 (60)
Head nurse	10 (36)
Supervisor	1 (4)
Others	0

#### 3.2.2. Correlations between measures

Correlations between outcome measures are shown in Table 3. All correlations were in the expected direction, although they did not always reach statistical significance in this small sample. For example, psychological distress demonstrated positive correlations with anxiety, depression, and post-traumatic stress disorder. Psychological flexibility was negatively correlated with anxiety, depression, and post-traumatic stress disorder, and positively correlated with subjective psychological wellbeing, such that higher psychological flexibility was, as expected, related to better outcomes.

**Table 3:** Correlations between measures at baseline (*n* = 28).

	**Distress (K6)**	**Psychological flexibility (CompACT)**	**Wellbeing (IWB)**	**Anxiety (GAD-7)**	**Depression (PHQ-9)**	**PTSD (PCL-C)**
Distress (K6)	1					
Psychological flexibility (CompACT)	−0.49^**^	1				
Wellbeing (IWB)	−0.82^***^	0.53^**^	1			
Anxiety (GAD-7)	0.73^***^	−0.31	−0.80^***^	1		
Depression (PHQ-9)	0.81^***^	−0.57^**^	−0.87^***^	0.86^***^	1	
PTSD (PCL-C)	0.86^***^	−0.54^**^	−0.83^***^	0.69^***^	0.83^***^	1

p, significance value; ^*^p < 0.05, ^**^p < 0.01, ^***^p < 0.001; n, number of subjects; k6, Kessler Psychological Distress Scale; CompACT, Comprehensive assessment of ACT process; IWB, Index of Well-Being; GAD-7, Generalized Anxiety Disorder; PHQ-9, Patient Health Questionnaire; PCL-C, PTSD Checklist – Civilian Version; PTSD, Post Traumatic Stress Disorder.

#### 3.2.3. Attendance and changes over time

We were unable to conduct post-intervention interviews with one participant (for personal reasons). Although the sample size was small, the changes in psychological stress, psychological flexibility, subjective psychological wellbeing, and depression after the 5-week intervention were statistically significant compared to the baseline level. We report our findings in Table 4. The K6 scores decreased by 27% from a mean of 10.19 (*M* = 4.63) to 7.44 (*M* = 5.22) (95% CI [−3.71, −1.78], *p* < 0.001). Psychological flexibility (CompACT) increased by 15% from 46.85 (14.86) to 53.74 (15.81) (95% CI [−12.35, −4.47], *p* < 0.001). The IWB scores increased from an average of 9.47 (*M* = 2.73) to 10.33 (2.42) (95% CI [0.07, 1.65], *p* < 0.05), an improvement of 9% in subjective psychological wellbeing. PHQ-9 scores decreased from 13.4 (5.1) to 4.2 (4.4) (95% CI [−2.78, −0.26], *p* < 0.05), reflecting an average improvement of 69% in depression.

**Table 4:** Comparison of pre-and post-assessment measures (*n* = 27).

**Outcome**	**Pre-assessment, Mean (SD)**	**Post-assessment, Mean (SD)**	**Mean difference**	**Percent change (%)**	**95% CI for Mean difference**	***p***
Distress (K6)	10.19 (4.63)	7.44 (5.22)	−2.74	−26.89	−3.71 to −1.78	< 0.001
Psychological flexibility (CompACT)	46.85 (14.86)	53.74 (15.81)	6.89	14.71	−12.35 to −4.47	< 0.001
Wellbeing (IWB)	9.47 (2.73)	10.33 (2.42)	0.86	9.08	0.07 to 1.65	< 0.05
Anxiety (GAD-7)	7.19 (5.38)	6.48 (5.24)	−0.70	−9.74	−1.90 to 0.49	0.242
Depression (PHQ-9)	9.15 (6.44)	7.63 (6.75)	−1.52	−16.61	−2.78 to −0.26	< 0.05
PTSD (PCL-C)	40.30 (14.92)	37.19 (15.92)	−3.11	−7.72	−6.73 to 0.51	0.090

n, number of subjects; SD, standard deviation; CI, confidence interval; p, significance value; k6, Kessler Psychological Distress Scale; CompACT, Comprehensive assessment of ACT process; IWB, Index of Well-Being; GAD-7, Generalized Anxiety Disorder; PHQ-9, Patient Health Questionnaire; PCL-C, PTSD Checklist – Civilian Version; PTSD, Post Traumatic Stress Disorder.

### 3.3. Phase 3: process evaluation

The semi-structured interviews with 28 participants (27 completers and 1 non-completer) were conducted after the 5-week pilot implementation.

Overall, the intervention program ran smoothly under the guidance and supervision of the facilitators. The participants indicated that the interventions could effectively alleviate their distress in many aspects, such as helping them to engage in the present moment, being aware of difficult emotions and thoughts, and improving their sleep. The main challenge was ensuring adherence to the intervention and ensuring that the prescribed dose was received promptly. Participants' main suggestions were to post the manual online as short videos to make it easier and more efficient, to have a brief and lucid introduction before each part to obtain a quick understanding of the goal of the following segment, and to modify the interface of the mini program.

#### 3.3.1. Participants' benefits from the program

In general, most of the participants considered the SH+ materials, such as the illustrated manual, the exercise audio, and the videos, very useful. The lessons and exercises in audio and short video formats were highly recommended. The participants indicated that they could comprehend and relate to the illustrations, and some reported that they had shared the illustrated manual with coworkers and patients in their unit.

Participants reported that SH+ assisted them in reducing distress, promoting relaxation, and enhancing their awareness and ability to be present. For example, one participant said:

*This program was very helpful. Especially the audio, a quiet relaxed state of mind occurred to me when I listened to instructions with music in the SH*+ *pack. There was a time when I was irritated and restless due to stress. I can't help getting annoyed with anybody around me. Then, I read the text and listened to the exercise audio on my telephone after work. I learned to live in the present, stop troublesome thoughts in my mind, and concentrate on important things. Surprisingly, I felt much better, forgot the distress, and kept a tranquil mind after reading, listening, and practicing (Female participant, 37 years)*.

Many participants also indicated that the skills learned from the program could help them deal with difficult thoughts and feelings and improve their sleep. Just as one participant said:

*This WeChat mini program was a great help. My sleep improved a lot, which was the most significant change I can tell. I don't have enough time for myself in the daytime because of busy clinical work. So, I usually used the applet before going to bed, which was relaxing. After listening to it, I can quickly fall asleep. This was the most obvious effect for me. By the way, I thought highly of your applet design because the audio can still play when the phone page was off which means there was no need to turn off the audio while I was almost falling asleep (Female participant, 36 years)*.

Apart from that, being aware of and re-recognizing distress, including physical symptoms and emotional responses to psychological distress, was a change identified by participants, as illustrated by the following quotes:

*After learning about this program, I realized that a certain amount of stress might cause some physical problems, including gastrointestinal reactions. When I was there (in the COVID-19 isolation region), my stomachache was so severe that I couldn't sleep at night. I had been suspected of having stomach problems and taking omeprazole all the time to kill the pain. After coming back, I had a gastroscopy immediately and it turned out fine. Now, I believe that it was stress and stomach cramps (Male participant, 36 years)*.

*I used to believe that blue mood or distress should be paid attention to only when it reaches a certain high level and becomes unbearable. However, after learning about this program, I found that some mild feelings and symptoms, such as irritability, sadness, inattention, etc., may be caused by some psychological distress, which was quite helpful for me to have a new understanding of distress. People tend to only notice mental health when they have symptoms or even illnesses, such as depression. The key is to notice when we don't (Female participant, 37 years)*.

#### 3.3.2. The difficulties of adhering to the program

Adherence to the self-help program was mentioned by most participants. Some participants stated that they tended to forget to practice the content or to do the exercises due to their intense and highly stressed clinical work, or distractions from social media on cell phones (e.g., WeChat, TikTok, Kuaishou, and RED). Several participants mentioned that it was difficult to persist with a 5-week course that indicated further adaptations, so the program fitted better with the busy lives of practicing nurses.

Changes in applet pushing strategies, such as increasing the push frequency of the mini program and adjusting the pushing time to the timetable of nurses, were proposed. For example, a head nurse said:

*I think learning the program through WeChat was the most convenient way. My distress and tension were eased while learning this applet, but most of the time, it was difficult to make time for practicing continuously. Maybe you can push the content a few times more for reminding. And it also could be helpful for learning when we receive lessons in our spare time, such as during noon break, off-work time, or time before bed (Female participant, 40 years)*.

Many participants assumed that overloaded information on a cellphone was not conducive to concentrating and insisted on learning the program. Learning in a variety of ways, such as brochures and sharing within groups, was identified by facilitators as illustrated by the following quote:

“*There are tons of mini-programs, public accounts, and information on the WeChat platform, which is hard for participants to focus on one on their own initiative or to adhere to it. According to this circumstance, they tended to pay attention to information that appears in front of them directly. It was suggested that several ways of learning to emphasize and go over the lesson, such as handing out simple brochures or conducting small group sharing regularly might be helpful.” (Female intervention facilitator, 26 years)*.

A few participants who did not complete the whole package of lessons in 5 weeks stated that they lost interest after the initial session.

“*I tried the exercise at the beginning, but it didn't attract me afterward. As for me, there was too much theoretical staff in the program making it obscure and abstract. I prefer more practical things or tools to help me out of difficult circumstances or problems specifically, such as relationship problems, relentlessness, anxiety, gloomy mood, or insomnia. A problem-based system of instruction and exercise would be much better.” (Female participant, 32 years)*.

## 4. Discussion

To the best of our knowledge, this is the first study in translation, adaptation, and initial evaluation of SH+ in China. Three main conclusions could be drawn from our findings. The series of methods we adopted were very necessary for the cultural adaptation of the entire set of interventions. Through expert group interviews and user testing, we made many adjustments to the language, semantics, context, and pictures in the guide based on expert feedback, making the guide more in line with Chinese culture and economic conditions. Compared to the traditional psychological intervention, the self-help intervention that relied primarily on pre-recorded materials was viewed as a significant advantage of the SH+ format in terms of fidelity and scalability, making stress management more accessible and allowing it to be applied to a large population without the need for psychological professionals. In the intervention period, many participants were not familiar with the content and operation methods, and some of them tended to miss part of the content due to work tension and other personal reasons. Other studies have found that active guidance by facilitators for the intervention was associated with larger effect sizes than unguided self-help interventions (19), and we feel some facilitation by peers could improve adherence in our context as well.

We found that it was necessary to make the program content more interesting and appealing by adding some scenes or stories close to life to grab the readers' continuous attention. In terms of intervention presentation, we found that short videos based on the WeChat program were a convenient and acceptable format. Short videos can be easily learned with full concentration in a limited amount of time. In addition, many nurses suggested that it was better to make the interface of the program classified by problems and symptoms, rather than by topic or chapter so that they could use the program according to their needs and it would be easier to maintain their interests and problem-solving capacity.

Importantly, the pilot showed promising results for SH+, which were consistent with studies of SH+ in other countries in the prevention and treatment of common mental disorders in refugees and asylum seekers (34–36). The cognitive interviews indicated that the adjusted guidelines were understandable, acceptable, and relevant. Our findings were consistent with the findings of related studies that mindfulness and acceptance and commitment therapy-based interventions are effective in reducing psychological distress and depression, as well as enhancing psychological flexibility and subjective psychological wellbeing among nurses (37–39). The change in anxiety and post-traumatic stress disorder was not statistically significant, which needs further evaluation through a sufficiently powered randomized controlled trial. These preliminary results suggest that the intervention program has a potentially positive effect on reducing the psychological distress of nurses working during COVID-19.

The main challenges we faced were poor adherence to the 5-week intervention cycle and the difficulty in producing the WeChat-based mini program. It may be that nurses were so occupied with the fast-paced, high-intensity, and stressful clinical nursing work, coupled with busy family life, that they forgot or missed the regular program lessons occasionally, which required facilitators to remind them constantly and push them repeatedly. In addition, competing information on various mobile platforms hindered our intervention from being easily used and made it difficult to stick to one program. Another challenge that needs to be considered was that most young people are immersed in short and quick information (especially short videos). Therefore, adapting the theoretical knowledge and skills into simple and short videos or texts is likely to be more attractive. Further adaptations catering to the specific context of nurses with busy professional and personal lives can enhance the value of the intervention.

Finally, a fully powered randomized controlled trial is required and is presently being planned for a more rigorous evaluation of the potential benefits of SH+ for Chinese clinical nurses.

## 5. Limitations

The following limitations must be considered when reading these conclusions. First, the evaluation of pre- and post-intervention scores in the pilot study was conducted with a limited sample and no control group. These results should not be interpreted as an indication of the effectiveness of the intervention but rather as an indication of the prospective feasibility and appropriateness of the intervention and the assessment measures. Future randomized controlled studies should be conducted to explore these trends in greater depth. Second, the analysis of the qualitative process evaluation data was initially conducted by one person, increasing the possibility of results that rely on the researcher's intuition and interpretative abilities and lack a broader and more complex understanding of the phenomenon. Finally, the main purpose of the qualitative study was mainly to explore the feasibility of the initial intervention program and the facilitators and barriers to the pre-experimental process, so the sample was drawn from a single source; as a result, the findings could not be generalized to a larger population.

## 6. Conclusion

Notwithstanding these limitations, our findings were promising and indicated that the translated and adapted Chinese version of SH+ was applicable and feasible to alleviate nurses' psychological distress in the Chinese cultural context. Cost-effectiveness is likely to be achieved when delivering an innovative, scalable psychological self-help intervention to large groups of participants in challenging settings. A randomized controlled trial as a more rigorous evaluation is also recommended to validate the effectiveness of the self-help intervention.

## Data availability statement

The raw data supporting the conclusions of this article will be made available by the authors, without undue reservation.

## Ethics statement

The studies involving human participants were reviewed and approved by the Ethics Committee of the Health Science Center at Xi'an Jiaotong University (No. 2020–1332). The patients/participants provided their written informed consent to participate in this study.

## Author contributions

LY and TT designed the study. XL and LY translated the materials into Chinese language. TT and ZH were responsible for the recruitment of participants. TT wrote the first draft. DW, AR, and XL critically review the article and gave their input. TT, LY, ZH, JS, YJ, and QG participated in the study design, data collection, and analysis. All authors revised and approved for submission.
