# Supplementary material for: Translation, adaptation, and initial evaluation of a guided self-help intervention to reduce psychological distress among nurses during COVID-19 in China
Source: Front Psychiatry. 2023 Aug 17;14:1168117. doi: 10.3389/fpsyt.2023.1168117 (PMC10469779; doi:10.3389/fpsyt.2023.1168117)
Supplement: Supplementary file 1 [file Data_Sheet_1.doc]

**APPENDIX 1 The instruction for cognitive interview**

**Procedure**

Groups will be conducted by: Asking the group to spend time reading through the book alone (or in pairs), collating feedback and then discussing as a group.

**Questions to consider:**

The aim of cognitive interviewing is to ensure the book is relevant, understandable and acceptable to nurses. Questions to consider include:

***Understandable***

*“How do you understand this text/picture?”*

*Are there any words, phrases, or images here that don’t make sense?*

*How would you change them to improve how they are understood?”*

***Relevance***

*“What relevance does it have to your community (or not)?”*

*How would you change it to improve relevance?*

***Acceptability***

*“How might people react to this exercise/image?”/ “Could someone become upset by this?” (e.g. could they become stressed, angry, offended).*

*Is it acceptable (i.e. non offensive) in your community?”*

*How would you make an improvement?*
